# Cloud-Based Machine Learning Methods for Parameter Prediction in Textile Manufacturing

**DOI:** 10.3390/s24041304

**Published:** 2024-02-18

**Authors:** Ray-I Chang, Jia-Ying Lin, Yu-Hsin Hung

**Affiliations:** 1 Department of Engineering Science and Ocean Engineering, National Taiwan University, No. 1, Sec. 4, Roosevelt Road, Taipei 10617, Taiwan; r05525122@ntu.edu.tw; 2 Department of Industrial Engineering and Management, National Yunlin University of Science and Technology, Yunlin 64002, Taiwan

**Keywords:** predictive maintenance, data communication, ensemble learning, process parameter, textile

## Abstract

In traditional textile manufacturing, downstream manufacturers use raw materials, such as Nylon and cotton yarns, to produce textile products. The manufacturing process involves warping, sizing, beaming, weaving, and inspection. Staff members typically use a trial-and-error approach to adjust the appropriate production parameters in the manufacturing process, which can be time consuming and a waste of resources. To enhance the efficiency and effectiveness of textile manufacturing economically, this study proposes a query-based learning method in regression analytics using existing manufacturing data. Query-based learning allows the model training to evolve its decision-making process through dynamic interactions with its solution space. In this study, predefined target parameters of quality factors were first used to validate the training results and create new training patterns. These new patterns were then imported into the solution space of the training model. In predicting product quality, the results show that the proposed query-based regression algorithm has a mean squared error of 0.0153, which is better than those of the original regression-related methods (Avg. mean squared error = 0.020). The trained model was deployed as an application programing interface (API) for cloud-based analytics and an extensive auto-notification service.

## 1. Introduction

In the textile industry supply chain, the industrial chain begins with upstream processes that involve the processing of petrochemical and natural raw materials into fibers. Moving to the midstream, fabric production occurs through processes such as spinning, weaving, and dyeing. The downstream sector primarily revolves around garment manufacturing [1]. According to a recent report, as of 2022, the textile industry contributed a substantial USD 1.2 billion, accounting for 93.2% of the worldwide textile industry output [2]. The global textile industry maintains a high level of competitiveness, with increasing demands for product quality and flexible production capabilities [3]. Textile manufacturing involves several processes, raw materials, and equipment settings. In the midstream of textile manufacturing, the raw material must pass through several manufacturing steps, including warping, sizing, beaming, weaving, and inspection. All of these factors can influence the quality of the product (Table 1) [4,5,6]. The first three steps mentioned above constitute the preparatory phase. In each phase, technicians adjust the production parameters of different textile machines according to the raw yarn characteristics. For example, setting the warp beam winding tension of the warping machine is essential in the preparation phase. When the tension is low, the yarn winding force is loose. If the tension is too high, the yarn will be stretched thin, and can easily break in subsequent processes. In general, the setting of process parameters depends on the staff operation, which leads to a time-consuming, resource-wasting, trial-and-error process. Furthermore, new technicians cannot easily learn from senior technicians the appropriate production parameter settings for different types of raw yarn on textile machines.

As digital transformation rapidly progresses [7,8,9], the manufacturing industry has evolving into a more cost-effective and value-enhancing business process by incorporating sensors, networks, and applications. Sensors are pivotal in transmitting substantial amounts of industrial data to the enterprise resource planning (ERP) system of the factory. Through self-learning, exploring valuable insights, and sharing with others, these data can significantly enhance the manufacturing process. The fundamental principle of digital transformation is value creation [10]. Thus, leveraging industrial data has proven to be an economical and efficient way to enhance business processes in the industry. This study aims to enhance the quality of the textile manufacturing process by using machine learning techniques to predict substandard products, thereby improving production efficiency and adding value to the existing digital infrastructure. This research not only uses machine learning with data but also enhances the computational performance through a query-based interaction approach. The midstream of the textile product manufacturing process serves as the use case. It employs manufacturing data and predictive models to forecast the quality of textile products using standard data analytics. The primary objectives of this study include intelligent production prediction and system design. This study highlights the top-performing predictive model, providing valuable insights to improve product quality. Additionally, the proposal introduces an online analytic module and a third-party application, facilitating the timely communication of analytics from machines to users. The contributions of this study can be summarized as follows:

- Investigated the prediction difference between the noninteractive and the interactive machine learning models.
- Developed an online analytical module for textile product manufacturing.
- Enhanced the value of the application by developing an office automation macro to enable automatic notifications.

The remainder of this study is organized as follows. Section 1 introduces the research background, research object, and contribution. Section 2 discusses related issues in the textile manufacturing industry. Section 3 outlines the data analytic procedure using the proposed approach and the system design for data communication. Section 4 details the experimental procedure, dataset used, and evaluation criteria. Section 5 presents the analysis results. Section 6 and Section 7 discuss and conclude this paper by describing the significance of the study, its limitations, and suggestions for future work.

## 2. Related Work

### 2.1. Textile Production Management

Textile manufacturing processes such as warping and sizing are essential for the manufacturing site. Exact production information and procedure control can enhance manufacturing efficiency [4,11,12]. Taking weaving as an example, it encompasses a variety of processes such as winding, warping, sizing, and drawing. This intricate journey involves calculations, production motions, and procedures, all of which converge to set the stage for the weaving process to unfold [12]. Drean et al. (2022) provided practical calculations in weaving preparation to ensure consistent quality and prevent failure of events during subsequent processing [4]. Bathrinath et al. used the fuzzy analytical hierarchy process to analyze problems in the yarn winding process and designed a corresponding strategy to improve productivity [11]. Recently, predictive maintenance has been applied in textile production management, including machines [13,14] and scheduling maintenance [15]. The current generation of smart factories integrates sensors and computer algorithms into the entire production line. These factories use IoT devices [16,17] to collect real-time data from physical and cyber spaces, leverage cloud computing [18] to handle big data [19], and employ artificial intelligence for statistical analysis, decision making, and planning. As a result, these smart factories can intelligently produce highly customized products. A new trend is the use of CPS [20,21], where software and physical components are deeply intertwined and can interact intelligently with each other. In the textile industry, the development of CPS began with the use of data analytics and sensor technology [22,23]. Through heterogeneous network connections, all entities, including physical equipment and software systems in smart factories, are connected to a common communication interface for data exchange and remote control. All raw data and value-added information are integrated into the ERP systems. On the basis of these emerging technologies, a CPS can recognize the states of physical entities in an industrial chain. Furthermore, it can generate cyber twins for these physical entities, replacing physical tests with more cost-effective, faster software simulations for prediction and management before implementing any planned or unexpected change. Ślusarczyk et al. reported that Industry 4.0 has constructively enhanced the efficiency of both production and services within the textile industry [24]. The constructive collaboration of CPS, interoperability, smart city integration, and innovative product intelligence collectively yield positive outcomes for both production and services [24,25]. The processing power of internet communication technology can be significantly increased, particularly when machine learning techniques are used to analyze existing data.

### 2.2. Data Analytics in the Textile Manufacturing Process

In recent years, the machine learning approach has been employed in the manufacturing process. Eltayib et al. used linear regression models to predict fabric tear strength in the warp and weft directions [26]. They revealed that the tear strength of the fabric in the weft was influenced by the tensile strength of the yarn and the yarn count, whereas the tear strength in the warp direction was impacted by the linear density of the fabric and the yarn count [26]. Lu et al. used an artificial neural network and multiple linear regression models to predict the tensile strength of individual wool fibers [27]. The coefficients of determination, obtained from their back propagation neural network and stepwise regression, highlighted a strong correlation between the measured and predicted strengths of wool, with an error value within an acceptable range [27]. A multilinear regression model and a geometrical method were employed to forecast sewing thread consumption for overedge stitches [28]. These studies demonstrated the effectiveness of these models in extracting valuable insights from data within the textile industry [26,27,28,29]. Hoque et al. applied the least absolute shrinkage selector operator (LASSO) to predict the bursting strength of single jersey cotton plain knitted fabrics [30], whereas Rabaca et al. used logit ridge regression and LASSO to predict business failure [31]. In a study by Gorgül et al. [32], a kernel ridge regression (KRR) model produced satisfactory anomaly detection results. Gorgül et al. recommended the use of KRR for modeling and anomaly detection, specifically in the context of temperature control in textile dyeing processes [32]. Their primary objective was to quickly identify issues with temperature controls, address failures in dyeing machines, and improve the efficiency of dyeing processes [32]. Ridge regression was used to predict the demand for textile products [33]. Although the algorithm was applied to textile manufacturing issues and demonstrated strong computational efficiency, its training mechanism is unidirectional and lacks interaction with the solution space. This limitation could lead to the emergence of local optimum problems. In contrast, recent advancements in reinforcement learning incorporate mechanisms for interaction with the environment. In this study, we introduce query-based learning. This approach enhances the predictive performance by fostering interaction with the solution space, validating with additional training instances, and exploring different solution spaces.

## 3. Materials and Methods

**Figure 1:**
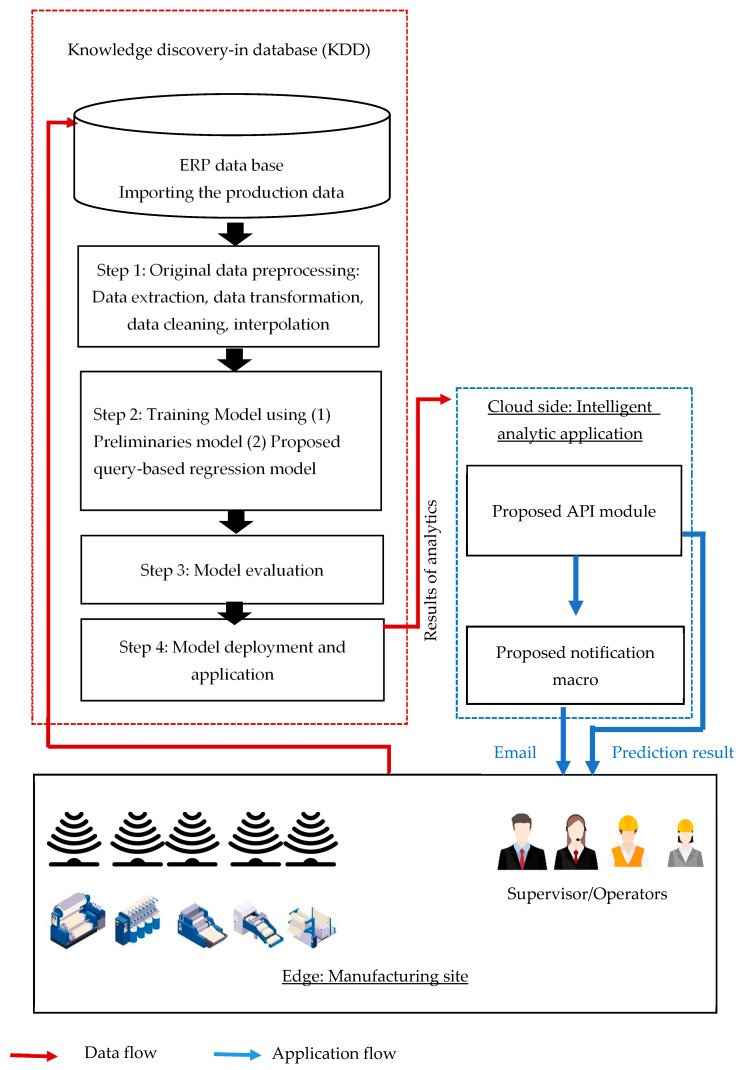
Flowchart of the data analytics of this study.

This study introduces a comprehensive workflow based on data from a case study. Edge data were transmitted to the ERP for data analytics and implementation notification. Machine learning approaches have proven to be highly accurate in certain instances. In this study, we used a regression-related method and our proposed method to predict issues in the textile manufacturing process. We processed the dataset according to the standard data workflow, the details of which are detailed in the experiment section. The process of knowledge discovery in databases involves data collection and preprocessing, modeling, model evaluation, and deployment (Figure 1). This approach can offer deeper insights into processes and allows exploration of the value of the imported data. The data are used to their fullest potential; both the original data and the results of the data analytics are transmitted to the API. Before a failure event occurs, the user receives an email notification and performs preventive maintenance.

### 3.1. Materials

**Figure 2:**
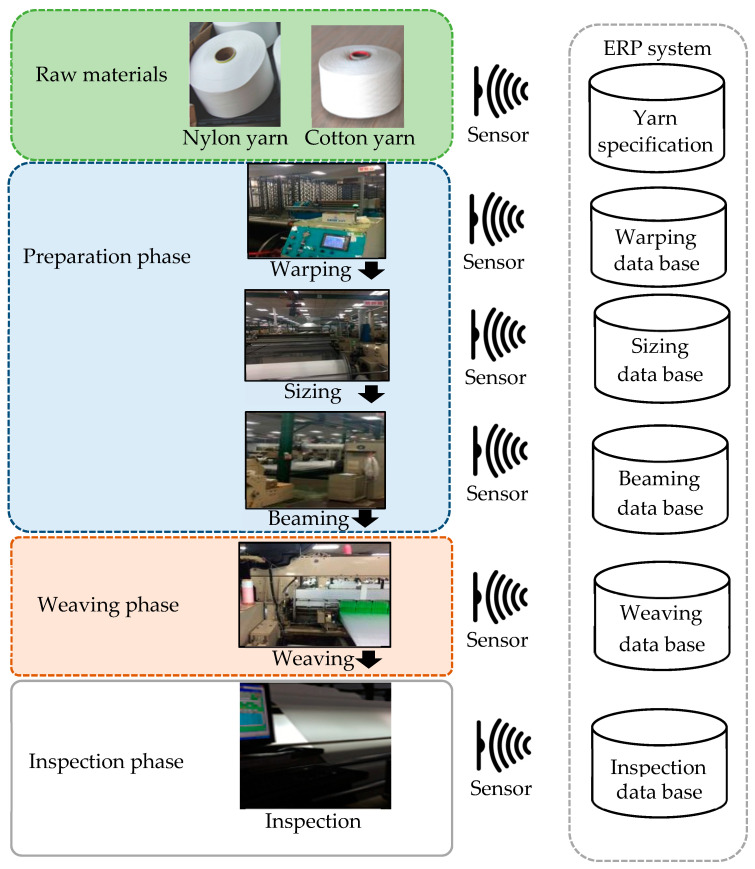
Data collection from textile manufacturing.

In addition to developing functional fibers, the use case was dedicated to developing interweaving technology and the use of equipment to blend natural and chemical fibers to weave functional blended fiber textile products. Accordingly, the aim of this study was to improve the quality of products using machine learning to analyze production parameter data. Figure 2 shows the textile production line in the use case. The production process can be divided into three major phases: preparation, weaving, and inspection. In each phase, technicians adjust the production parameters of different textile machines according to the characteristics of the raw yarn, and the sensors transmit parameter data to the ERP system database.

In this study, the data were obtained from the ERP system, and the data format was a .csv type file. The obtained data were analyzed for early production diagnosis in textile manufacturing. Table 2, Table 3 and Table 4 describe the dataset and the specific prediction target. The data were obtained from the textile manufacturing process, encompassing its crucial phases, such as warping, sizing, beaming, and weaving. The original data had to be preprocessed. The different preprocessed datasets transformed into sixteen essential production attributes and thirteen raw material attributes were imported into the prediction model (Table 3 and Table 4).

### 3.2. Data Preprocessing

The company under study operates service factories worldwide, with textile machines in each factory using sensors to gather production data. This sensor-collected data, along with the production line information from each factory, are uploaded to the company’s ERP system for centralized management. The dataset used in this study was collected from the ERP system of the factory under investigation. The data source comprised five datasheets: warping, sizing, beaming, weaving, and inspection. These datasets contained 37, 46, 31, 25, and 12 columns, with 26,103, 15,950, 37,089, 161,821, and 481,610 records, respectively. The original data contained missing values and complicated content, and the weaving data were inherently complex [34]. Unclean or incorrect data can severely impact subsequent model training and prediction. Therefore, it was crucial to clean the original data [35] and identify their attributes using the extract–transform–load (ETL) method. ETL is a data preprocessing approach in which the original data are corrected, cleaned, and formatted. In this study, we extracted the data based on recommendations from senior experts in the production line of the factory under study. The main and frequently used attributes were extracted from the five datasets to form a new dataset. The extracted data were cleaned by removing incorrect, incomplete, malformed, or redundant data from the datasets. The cleaned data were then transformed into a machine-readable format. The preprocessing steps used in this study are as follows:

(1). Missing value process: The original dataset contained missing values and redundant columns (e.g., remark column). In this study, we filtered out data with missing values and removed redundant columns. The weaving machine could interweave a maximum of four yarns, resulting in many empty columns (variables) in the integrated dataset. For instance, when the weaving machine interweaves two yarns, the columns for the third and fourth production lines in the preparation phase are left empty.
(2). Feature interpolation: Blended fabrics are woven with different warp yarns, and each yarn material is assigned the same job order number. As a result, there are multiple rows of data with the same job order number, each representing a different set of operational parameters for these yarn materials during the preparation phase (warping, sizing, beaming). In this study, given a maximum of four different warp yarns, the original raw data may have contained empty values. Circular interpolation seamlessly interpolates cyclic numerical values. The main objective of circular interpolation is to achieve smooth, continuous interpolation within a numerical set, ensuring a steady trend in a constantly evolving environment. In this study, we filled the empty columns using circular interpolation, adhering to the practical production line schedule rule, as shown in Table 5. We also used circular interpolation to fill in the other empty values.
(3). Data transformation: The original data values were a mixture of descriptions and number values. Therefore, we used regular expressions (regex) to split the aggregated attributes of the production data [e.g., “0010/25N wine red“]) into individual attributes. For instance, the yarn specification attribute was the aggregate of three independent attributes: denier, fiber base, and material.
(4). Outlier Removal: The original data required the outlier removal process, and this study filtered out data beyond three standard deviations in each column.
(5). Normalization: We used min–max scaling to adjust the numerical range of each column between 0 and 1.

### 3.3. Preliminary Models

In this study, we employed various regression algorithms to predict the production quality. Linear regression served as the foundation method for predicting the target variables. The LASSO addresses overfitting by eliminating redundant features, whereas ridge regression removes unnecessary features. Elastic net regression combines both strategies for enhanced prediction. Each regression algorithm has its own advantages and disadvantages, and we explored the differences in the prediction results of these regression methods in this case study.

#### 3.3.1. Linear Regression

Linear regression is a productive linear approach for modeling the relationships between independent and dependent variables [36]. As a data-driven technique, linear regression identifies a linear equation to predict the positions of future data points. In the context of two-dimensional data, the regression equation appears as a line. The difference between the existing data points and the line of the equation is the error function. The core of regression machine learning algorithms is to minimize this error function by determining the optimal equation. A common error calculation method is the least squares approach, which strategically minimizes the sum of squared errors.

**Figure 3:**
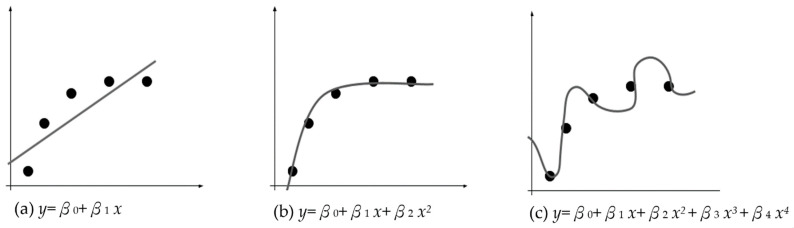
Linear regression’s three (3) main phenomena. Note: The mathematical relationship between two variables *x* and y, where β_0_, β_1_, β_2_, and β_3_ are coefficients.

The conceptual underpinning of this algorithm is illustrated in Figure 3 within a two-dimensional framework. Linear regression is used to derive a linear fitting equation. The error is the measured distance between the line and the data points. The primary goal is to minimize this error and identify an equation that closely aligns with the emerging data trend. This resultant equation forms the basis of the linear regression model. When new data points are introduced, the independent variable is input into the model, producing the dependent variable (the predicted value). During training, linear regression algorithms often face issues of underfitting and overfitting. Underfitting occurs when the equation does not adequately minimize the errors with the existing data points (Figure 3a). Conversely, overfitting occurs when the equation overly conforms to the existing data points, failing to capture the true data trend and resulting in substantial errors when new data inputs are introduced (Figure 3c). Figure 3b illustrates the ideal scenario. Ridge regression and LASSO were introduced to address these issues. Both methods operate by introducing an appropriate penalty term to shrink the model’s feature parameters, a technique known as regularization, to minimize the error between the predicted and actual values.

#### 3.3.2. LASSO Regression

LASSO is a regression analysis algorithm [37,38] that employs regularization and variable selection to enhance the prediction accuracy [37]. This is a refined version of linear regression designed to counter potential overfitting in multiple linear regressions. The L1 penalty model inherent in the LASSO regression encourages specific feature coefficients to approach zero, introducing a sense of sparsity within the feature set. LASSO regression strategically uses the L1 penalty for feature selection, effectively addressing overfitting concerns in multiple linear regressions. LASSO is more effective for models with fewer features, resulting in streamlined and less complex structures.

#### 3.3.3. Ridge Regression

Ridge regression estimates the correlations between the predictor and observation variables [39]. Ridge and LASSO differ significantly; the key distinction lies in how they allocate penalties to feature coefficients. Ridge regression introduces a penalty term in the least squares sum, categorizing it under the category of L2 penalty models, whereas LASSO regression employs an L1 penalty term.

#### 3.3.4. Elastic Net Regression

Elastic net regression integrates LASSO and ridge regression to address issues such as multicollinearity and overfitting in linear regression models. This versatile technique in statistics and machine learning offers a dynamic solution to common regression challenges. LASSO enforces sparsity by pushing some coefficients to zero, whereas ridge regression penalizes large coefficients using a squared term. In contrast, the elastic net introduces a unique penalty term that encompasses both L1 (LASSO) and L2 (ridge) regularization. The strength of elastic net regression is its nuanced loss function, which combines the sum of squared errors with penalties from both L1 and L2 regularization. This balanced integration of penalties enables elastic net regression to balance feature selection (sparsity) and coefficient shrinkage. This is particularly useful in scenarios with many correlated features, where a nuanced level of feature selection is crucial. In summary, elastic net regression is a versatile and sophisticated approach that combines the strengths of LASSO and ridge regression to provide a comprehensive solution for regularization in linear regression models [40]. Van (2023) found that elastic net regression exhibits a strong ability to predict nitrogen concentration [41].

### 3.4. Proposed Query-Based Regression Model

**Figure 4:**
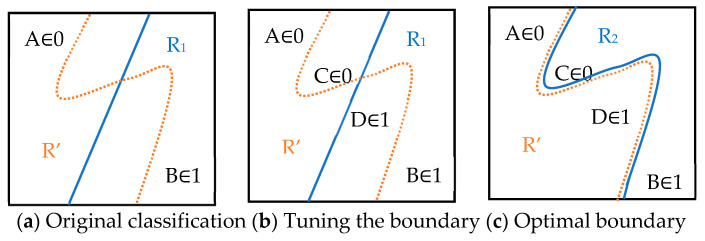
Query-based learning concept. Note: (**a**) displays the original classification. R1 is the predicted classification boundary, and R′ is the actual boundary, with A and B representing the data points. As illustrated in (**b**), query-based learning can select additional data points near the current boundary (e.g., data points C and D) to establish a better classification boundary. Finally, as shown in (**c**), the new predicted classification boundary R2 can be obtained.

Exiting machine learning algorithms have demonstrated good computation performances in real case studies. However, some of these algorithms are one-direction procedures from beginning to end [36,37,39,41], and the training models lack bi-direction interaction with the environment. In recent years, generative methods and interactive reinforcement methods have been applied to existing machine learning models to improve their computational performances [42,43,44]. This study proposes query-based learning, which takes advantage of the generative and interactive reinforcement methods by creating new learning instances and bi-direction interactions in the solution space. Query-based learning [45,46,47,48,49] is an active learning technique that effectively enhances the model performance by incorporating key data points. Figure 4 illustrates the concept of query-based learning using a simple binary classification example. The initial training dataset contains data points A and B from two different categories. A classification boundary R1 is obtained, and the actual classification boundary is R, as shown in Figure 4a. Query-based learning can select additional data points near the current boundary (e.g., data points C and D) to establish a better classification boundary, as shown in Figure 4b. By querying the oracle, the query-based learning identifies the categories of C and D and can then train a new classification boundary R2, as shown in Figure 4c. Query-based learning employs a learning protocol to dictate the method of information accumulation.

**Figure 5:**
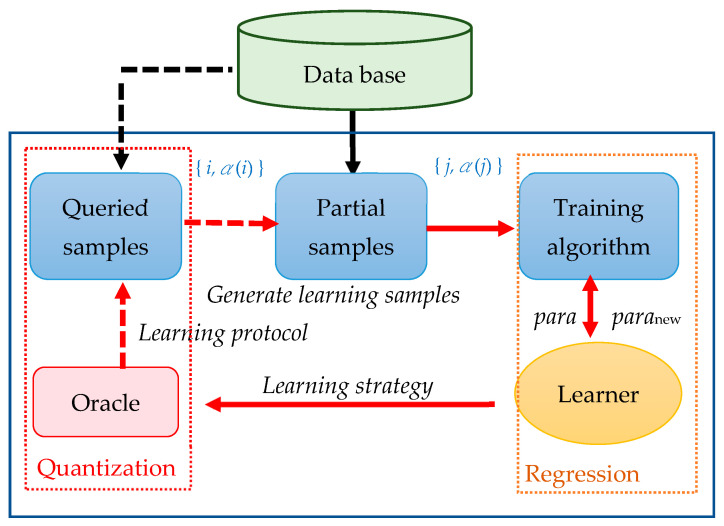
Procedure for the proposed query-based method in regression.

Query-based learning provides a deduction procedure for the training model to correct the computing path and improve the solution-exploring performance of each machine learning model during training. Under the learning protocol of query-based learning, the training model does not passively recognize correlations between data. Instead, it actively learns from a controlled environment by observing and recording the computation results (e.g., evaluation criteria). The query-based approach provides the learning protocol for the machine learning model. Input data can take the form of examples that illustrate the concept to be learned or oracles that indicate whether the data exemplifies the concept. Thus, the training model has the flexibility to use not only existing samples, but also additional samples generated by the oracle to enhance the training model. With the data point of the query set as Q, the oracle responds with Res(Q). The pair (Q, Res(Q)) refers to the queried sample (Figure 5). The sample query method is an incremental approach that dynamically adjusts the sample size extracted from each class [50]. We assume that the learner has the autonomy to inquire about the training samples based on a specific rule, rather than relying on random selection; training samples from decision boundaries yield the best training results. We set point Q such that Res(Q) = 0.5. In the proposed method, we first examine untrained samples to determine whether they are classified correctly. Because the output also indicates the probability of making a correct prediction for the samples, we can easily store these correctly classified samples in a priority line (max-heap). The stored points with the correct predictions are then selected as additional training samples. The learning process is considered complete when either the number of iterations exceeds the given threshold N or the obtained RMSE is below the given threshold RMSE. Figure 5 illustrates the framework of the query-based regression. The step-by-step process of the proposed algorithm is as follows; learners are classified into two types: strong learners (instances with low prediction error) and weaker learners (instances with high prediction error):

- Step 1. Initialize the parameters within the regression model using a random configuration. Let the iteration threshold be N and set the error threshold as the RMSE.
- Step 2. Configure the dataset as D = {Res_i_ ∈ Sample^n^}, where n is the number of selected attributes. The data point of the query is set to Q, and the oracle responds with Res(Q). Collect the partial training sample set DD ⊂ D through stratified random sampling.
- Step 3. Train the regression model employing the training sample set DD. If the error E is below the RMSE or the iteration number exceeds N, exit.
- Step 4. The untrained sample set (D − DD) must be analyzed.
- Step 5. According to the two learning strategies, by adding samples to the DD, the training sample set consists of either (a) samples of the most correct prediction, which is learning from the strong learners [47,49], or (b) samples of the least correct prediction, for which the weak learners are used to explore the fuzzy or unexplored solution spaces [48].

The original regression training is one-directional, import data training with an algorithm and a penalty model. Figure 5 demonstrates the interactive ability of query-based learning and the training model being dynamically adjusted according to each iteration outcome. The proposed method can improve the existing model using a self-learning mechanism.

### 3.5. Model Deployment and Application

**Figure 6:**
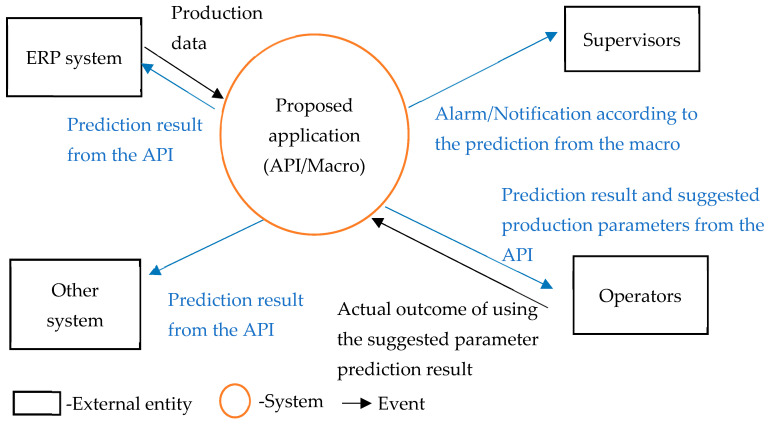
Diagram of the system context for the final stage.

Further use of data is crucial for data-driven digital transformation at manufacturing sites. With the rapid development of the IoT and related applications, various types of third-party resources are widely adopted in different cocreation value applications. We deployed the trained model as an API and used a Django-based API server for other applications to access the model using the RESTful format. We developed an email macro to enhance the efficiency of production management. Upon receiving an anomalous product quality prediction, the user can automatically send a reminder email to their staff. Algorithm 1 shows the procedure of the proposed approach. Temporary cursors and counters were used to implement the algorithm. On the basis of the system analysis and design theory, we prebuilt the system context diagram, as shown in Figure 6.
**Algorithm 1** Anomaly prediction notification**Description:** The API exports the result as a worksheet. The cells of the worksheet can be used in more applications, such as predictive maintenance. While users receive the predicted result from the cells of the worksheet and reach the threshold value, the proposed macro can automatically send notifications to other staff. **Procedure** AlarmNotify (date_cursor, attriValue, attriThres)1:**Initialize:**2:Let emailAddrQueue be a queue of email address items3:Let date_cursor be a temporary cursor of the currently used resource4:Let sysDate be the current date time of running the algorithm5:Let attriValue be the value of the target attribute6:Let attriThres be the threshold value of the target attribute7:sysDate = the current system date8:   **if** (date_cursor = null) then9:    dat_cursor ← sysDate10: **endif**11:**Begin:**12: **foreach** Item of emailAddrQueue do13:  **if** (attriValue ≥ attriThres) then14:   date_cursor ← sysDate15:   form the notification content16:   execute the automatic notification17:  **endif**18: **endfor**19:**End Procedure**

## 4. Experiment

**Figure 7:**
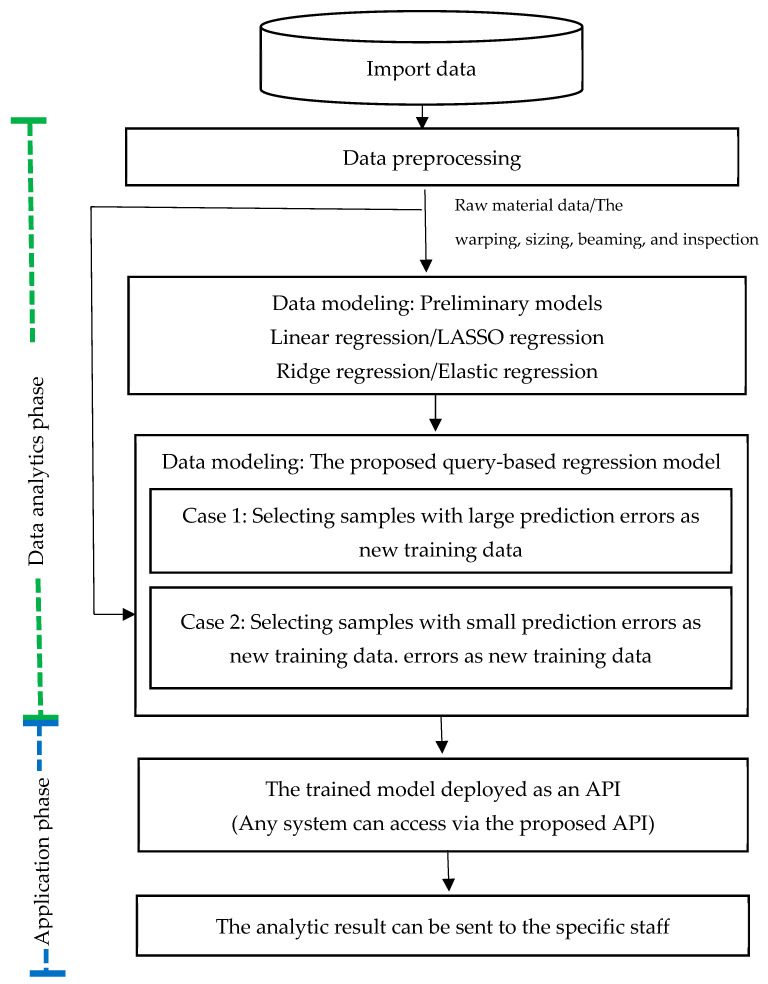
Procedure of the experiment.

The dataset source for the experiment was the ERP system used in the proposed use case. The dataset involved textile manufacturing processes such as warping, sizing, and beaming. Our aim was to forecast the quality of the product in the textile manufacturing process. In this study, we used the quality of the finished product to tune the solution space of the model. Figure 7 shows the experiment flow, which consisted of two phases: the data analytic phase and the modeling phase. First, the dataset needed to be preprocessed and modeled. This study compared the proposed method with the preliminary methods. Predictive modeling was implemented using various regression techniques, including linear, LASSO, ridge, and elastic net regression. The preprocessed data were imported into the proposed query-based model. Finally, the trained model was deployed as an analytic API that could be integrated into the cloud system for further use, such as for early notification in cases with anomalous predictive outcomes.

### 4.1. Evaluation Criteria

We used the mean squared error (MSE) to evaluate the prediction performance. The evaluation criteria are described below. The MSE (Equation (1)) is a pivotal tool for error estimation; it gauges the disparity between predicted and observed values. In this study, The MSE is a loss function that offers a robust means of estimating and evaluating prediction performance:

(1) $$MSE=\frac{1}{S}\sum_{i=1}^{S}{\left({var}_{i}-{var\prime }_{i}\right)}^{2}$$

where a vector of m predictions is generated from a sample of s data points for all variables, var is the observed value of the predicted variable, and var’ is the predicted value.

We used the improvement rate, which compares the original model and the proposed method (Equation (2)), as a criterion to evaluate the performances of the algorithms:

(2) $$\;\mathrm{improvement}\;\mathrm{rate}=\frac{{\mathrm{MSE}}_{proposed}-{\mathrm{MSE}}_{original}}{{MSE}_{original}}$$

### 4.2. Validation

K-fold cross-validation methodology is commonly used to compare and select predictive models. This involves iteratively resampling a dataset and recursively dividing the original set into multiple subgroups. This study employed 10-fold cross-validation to assess the trained model while mitigating potential biases stemming from data partitioning. The overarching concept of 10-fold cross-validation is the recursive partitioning of a dataset, culminating in the computation of average values across these partitions. The procedure validation procedure is as follows [51].

(1). Data Division: The initial dataset is segmented into 10 subsets or folds, each maintaining an approximately uniform size.
(2). Model Training and Assessment: The model undergoes 10 training cycles, where each training session employs nine folds for training and reserves one fold for validation. During the initial iteration, the model is trained on Folds 2 to 10 and evaluated on Fold 1. Subsequently, in the following iterations, training encompasses Folds 1 and 3 to 10, with evaluation on Fold 2, and so forth. This process repeats until each fold serves as a validation set precisely once.
(3). Calculation of Performance Metrics: Evaluation criteria are computed for each iteration, generating 10 distinct performance scores.
(4). Compilation of Overall Performance Metrics: The 10 performance metrics are commonly averaged, yielding a singular performance score that encapsulates the model’s comprehensive performance. This average performance score provides a more resilient estimate of the model’s anticipated performance on unseen data.

## 5. Result

This section shows the predictive results of the regression-related approach and the proposed query-based approach in our case study; 60% of the preprocessed dataset was used as the training dataset, and 40% of the preprocessed dataset was used as the testing dataset. In this study, 10-fold cross-validation was used to evaluate the trained model to avoid possible bias from data segmentation. A total of 28 training attributes comprised yarn specification, warping, sizing, beaming, and weaving, and one predicted target was the quality rate of the product. In the model setting, the LASSO regression was trained with L1 regularization, and the alpha parameter value was set to 1. The ridge was trained with L2 regulation, and the alpha parameter value was set to 1. The elastic net regression model was trained with L1 regularization with alpha = 1.0. The MSE was the estimated error between the real quality variable and the predicted variable. The average MSE value of the original regression (Linear/LASSO/Ridge/Elastic Net) was 0.02, and that of the proposed method was 0.0153. Compared with unused query-based learning, the regression model that used query-based learning had an improvement rate of 22%. This result is beneficial for predictive maintenance in manufacturing (Table 6).

**Figure 8:**
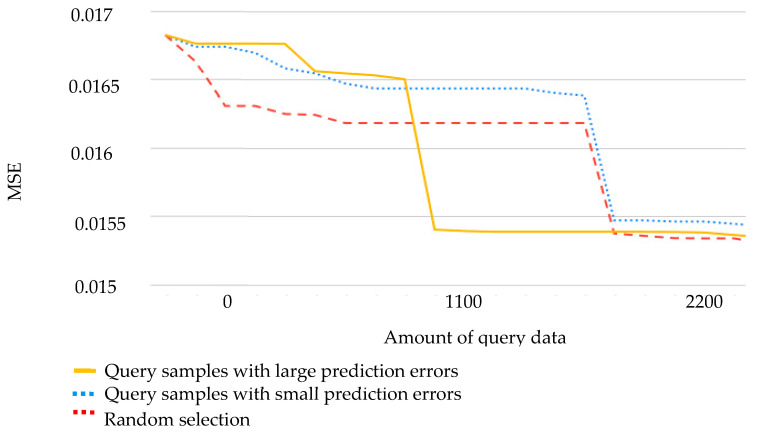
MSE of query-based regression in production quality prediction.

Query-based learning is an active learning technique that effectively enhances the overall performance of a model by retraining it with the addition of new data. We compared various learning strategies using the following procedure: from 5898 data points, 60% were selected as the training data, and the remaining 40% served as the testing data. New data suitable for learning were identified from the neighboring points of the testing data. Two learning strategies were employed to generate new data in this experiment: selecting samples with large prediction errors as new training data and selecting samples with small prediction errors as new training data. Two learning strategies and a nonuse strategy (randomly generating new training data) were used in the experiment. The ability of the three methods to strengthen model prediction in query-based learning was compared. The results indicate that providing learning guidance by retraining using poorly performing samples yielded the best outcomes (Figure 8).

**Figure 9:**
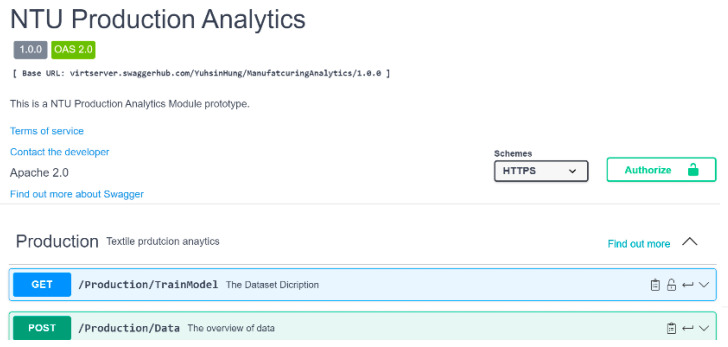
The trained model was deployed as an API for further use.

The trained model was deployed as a RESTful API that can be used in edge- or cloud-based analytics for the prediction of process parameters. The process of an API call starts when it is transmitted from the client side to the API endpoint. This endpoint, which is the final destination, is mainly found in web applications and servers. Consider, for example, a mobile client that initiates an API call. This call travels toward the specified API endpoint, which is often represented by the server. Upon reaching the server side, an API call undergoes a sequence of actions: it is processed, a request is executed, and a response is dispatched back to the client side. This dynamic interaction constitutes the essence of API calls and allows communication between the client side and the API endpoint. In this study, submission was detected by the server and automatically imported into the trained model based on the API message (Figure 9). Next, the server sends the predicted results to the client as a response.

**Figure 10:**
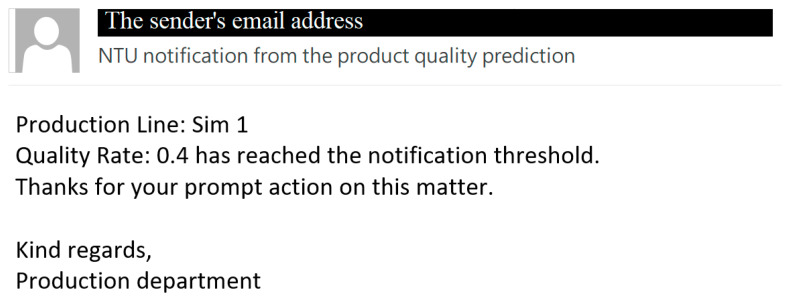
Result of early anomaly notification.

Numerous ERP systems offer features that enable users to export data seamlessly to Excel, thus opening avenues for in-depth analysis, customized reporting, and data manipulation. This is particularly beneficial for individuals who find Excel more intuitive for specific tasks. In contrast, most ERP systems facilitate data importation from Excel files. This functionality is valuable when users need to perform bulk updates or input data using the familiar, user-friendly interface of Excel for data preparation. In this study, we used Visual Basic for Applications, which is an office automation tool that automatically executes Excel’s features using macros. The analytic results are then exported as a worksheet for further use. After receiving the predicted anomalies from the worksheet, a user can automatically send a notification to other staff. Figure 10 shows how emails are correctly sent to the user using the proposed anomaly notification macro.

The main finding of this study is that the proposed method can predict product quality with promising advantages. Linear regression showed a better prediction performance than the other algorithms for product quality prediction. According to previous studies, query-based learning exhibits an excellent computing performance because of its advantages in generating new learning samples and training the model with different parameters [42,43,44,45,46,47,48,49,50]. This study obtained similar results. The proposed query-based regression model can search a larger solution space than the original preliminary function [42,43,44,45,46,47,48,49,50]. Thus, it exhibited a good prediction ability in the case study. Finally, the trained model can be widely applied for further use through the API and notification macro. The seamless flow of transmission data streams, which facilitates communication from edge-side equipment to cloud-side networks, is a pivotal catalyst for the continuous progress of the IoT. This interconnected data exchange not only forms the backbone of the IoT infrastructure, but also acts as a driving force behind its ongoing evolution [49]. This study fulfills the communication requirement between edge-side equipment and cloud-side applications. The efficacy and efficiency of the proposed API and macro fundamentally contribute to the dynamic landscape of the IoT, propelling advancements in data analytics and real-time decision making. Future studies will be conducted to analyze different data sources from other textile manufacturing processes, computerized machines, and manufacturing execution systems. This information will help determine the correlation between product quality and different manufacturing properties. Furthermore, the developed approach will be implemented and validated in an industrial case study and smart maintenance case studies to demonstrate its efficiency and capacity to support maintenance engineers and product inspectors.

## 6. Discussion

One key transformation for textile factories is their move to fine textile products, which usually means high quality, high customization, and delivery in a short time. However, the trial-and-error method for determining proper production parameters is time consuming and resource wasting. The proposed method has promising advantages in predicting product quality in textile manufacturing. Previous studies have stated that query-based learning leads to excellent computing performances because it has the advantage of tuning models to evolve dynamically [45,46,47,48,49]. The current study obtained similar results. The proposed query-based regression model searches a larger solution space than the original preliminary function [47,48], thus exhibiting a good prediction performance in our case study. The trained model can be further used through the API and notification macro. In this study, the seamless flow of transmission data streams facilitates communication from edge-side equipment to cloud-side networks. This interconnected data exchange constitutes the backbone of the IoT infrastructure and serves as a driving force behind its ongoing evolution [16,17,18,52].

### 6.1. Limitations of the Study

This study focused on investigating the influence of using the proposed query-based learning method with regression-related methods. In addition, the sizes and attributes of the industrial datasets limit this study. However, each industry has its own unique facilities, devices, products, and manufacturing processes. The study case was a midstream textile product manufacturer. The more manufacturing scenarios considered in a production line, the more data acquired in a factory.

### 6.2. Future Study

In the cloud-based environment, safeguarding sensitive manufacturing data in the realm of data security poses numerous challenges for organizations. The primary concerns revolve around the potential for data breaches arising from weak authentication and compromised credentials. Additional threats emerge from inadequate access controls and poorly configured permissions, which may result in unauthorized access to critical data while the user uses the API to access data analytics. Therefore, the proposed API uses authorized login account validation. In the future, API access will use the single-sign-in method to reduce the risk of unauthorized access, and the data value can be encrypted for data privacy protection. In addition, the uploaded data overview and confirmation can address incorrect data injection attacks, which may influence the prediction results.

## 7. Conclusions

For complex textile manufacturing, the production parameter setting in the manufacturing process is based on the operator’s experience or trial-and-error testing. Human error may influence the quality of the product. To improve production efficiency, this study proposes a case study that demonstrates how to learn from existing data and integrate them as an application service into existing cloud infrastructure. This study has two findings: data analytics and system design. From the viewpoint of data analytics, we used query-based learning to reinforce model training. The proposed query-based method exhibits better prediction than the original model in high-quality production manufacturing because the proposed method adjusts the solution space based on dynamic integration with the solution space. From the viewpoint of system design, the trained module was deployed as an API for online analytics and further use. To add value to the existing environment, this study proposed an API that can be integrated with office automation. Thus, we developed an auto-notification macro for the user to obtain notifications when a predicted anomaly event occurs. Finally, the case results show that the proposed approach can preventively assist in product quality maintenance using data analytics and cloud technologies.

## Figures and Tables

**Table 1:** Description of the primary manufacturing process.

Procedure	Action	Description
1	warping	The process of arranging and aligning yarns or threads in parallel to form the lengthwise foundation of a woven fabric [4,6].
2	sizing	The process of applying a protective substance, known as sizing, to warp yarns before weaving [4,5,6].
3	beaming	The process that follows the sizing process is part of the preparation of warp yarns for weaving [4,6].
4	weaving	The process of interlacing two sets of yarns at right angles to create a fabric [6].
5	inspection	The process of identifying and rectifying defects in the finished product early in the production stage.

Note: Warping, sizing, and beaming are integrated in the preparation phase.

**Table 2:** Manufacturing dataset.

Filename(.csv)	Columns	Raw	Description
warpop	37	26,103	production parameters in the warping process
sizeop	46	15,950	production parameters in the sizing process
beamop	31	37,089	production parameters in the beaming process
weaveop	25	161,821	production parameters in the weaving process
Inspection	12	481,610	fabric inspection results

**Table 3:** Raw material parameters.

Raw Material (13 Attributes)	Name	Description
Yarn Specification (13 attributes)	TOTALLENGTH	Actual length of warp ^1^
	THEORYLENGTH	Theoretical total length warp ^1^
	WARPTOTAL	Number of warps ^1^
	YARNSPECDENIM	Denier ^2^ number of yarn specifications
	YARNSPECFIBERBASE	Fiber number of the yarn specifications
	DENIM	Theoretical denier ^2^ number of yarn specifications
	FIBERBASE	Fiber number of the yarn specifications
	UNITWEIGHT	Weight per unit
	GRANULARITY	Granularity of the yarn
	WARPLENGTH	Length of the warp
	WARPSTRIP	Length of beaming
	WARPLENGHT	Length of warping
	SIZINGLENGTH	Length of sizing

Notes: ^1^ Warp refers to the set of yarns or threads that run lengthwise in a woven fabric. ^2^ Denier is a unit of measurement used in the textile industry to express the linear density of fibers or yarns.

**Table 4:** Equipment setting parameters.

Process (16 Attributes)	Name	Description
Warping (5 attributes)	WARPSPEED	The speed of warping
	WARPPRES	The tension of the Warper’s Beam
	SSTENSION	The tension of monofilament
	WARPTENSION	The tension of warping
	HYDRATENSION	The tension of hydraulic warping.
Sizing (6 attributes)	SIZINGSPEED	The speed of sizing.
	SIZINGBPRES	The pressure of sizing
	SIZINGATENSION	The tension of sizing (roll out)
	SIZINGBTENSION	The tension of sizing (winding)
	CONSISTENCY	The density of forming polymeric material
	DENSITY	The density of sizing
Beaming (4 attributes)	BEAMSPEED	The speed of beaming
	BEAMATENSION	The tension of roll-out
	BEAMBTENSION	The tension of winding
	BEAMTENSION	The tension of beaming
Weaving (1 attribute)	WEAVEBTENSION	The tension of weaving
Inspection (1 attribute)	QUALITYRATE	The value with range 0 to 1 1 = high product quality

**Table 5:** Filling rules for different degrees of interweaving.

Degree of Interweaving	Integrated Dataset	Fill Rules and Final Dataset
1	{A, 0, 0, 0}	[A, A, A, A]
2	{A, B, 0, 0}	[A, B, A, B]
3	{A, B, C, 0}	[A, B, C, A]
4	{A, B, C, D}	[A, B, C, D]

Note: A, B, C, and D are the IDs of the production line.

**Table 6:** Prediction results for the production quality.

Model	MSE
Linear Regression	0.0211
LASSO Regression	0.0191
Ridge Regression	0.0193
Elastic Net Regression	0.0190
Query-Based Regression	0.0153

## Data Availability

No new data were created or analyzed in this study. Data sharing is not applicable to this article.
